# Real-time liver tumor localization via combined surface imaging and a single x-ray projection

**DOI:** 10.1088/1361-6560/acb889

**Published:** 2023-03-09

**Authors:** Hua-Chieh Shao, Yunxiang Li, Jing Wang, Steve Jiang, You Zhang

**Affiliations:** 1 The Advanced Imaging and Informatics for Radiation Therapy (AIRT) Laboratory, The Medical Artificial Intelligence and Automation (MAIA) Laboratory, Department of Radiation Oncology, University of Texas Southwestern Medical Center, Dallas, TX 75390, United States of America

**Keywords:** liver, real-time tumor localization, x-ray, surface imaging, deep learning, biomechanical modeling

## Abstract

*Objective*. Real-time imaging, a building block of real-time adaptive radiotherapy, provides instantaneous knowledge of anatomical motion to drive delivery adaptation to improve patient safety and treatment efficacy. The temporal constraint of real-time imaging (<500 milliseconds) significantly limits the imaging signals that can be acquired, rendering volumetric imaging and 3D tumor localization extremely challenging. Real-time liver imaging is particularly difficult, compounded by the low soft tissue contrast within the liver. We proposed a deep learning (DL)-based framework (Surf-X-Bio), to track 3D liver tumor motion in real-time from combined optical surface image and a single on-board x-ray projection. *Approach*. Surf-X-Bio performs mesh-based deformable registration to track/localize liver tumors volumetrically via three steps. First, a DL model was built to estimate liver boundary motion from an optical surface image, using learnt motion correlations between the respiratory-induced external body surface and liver boundary. Second, the residual liver boundary motion estimation error was further corrected by a graph neural network-based DL model, using information extracted from a single x-ray projection. Finally, a biomechanical modeling-driven DL model was applied to solve the intra-liver motion for tumor localization, using the liver boundary motion derived via prior steps. *Main results*. Surf-X-Bio demonstrated higher accuracy and better robustness in tumor localization, as compared to surface-image-only and x-ray-only models. By Surf-X-Bio, the mean (±s.d.) 95-percentile Hausdorff distance of the liver boundary from the ‘ground-truth’ decreased from 9.8 (±4.5) (before motion estimation) to 2.4 (±1.6) mm. The mean (±s.d.) center-of-mass localization error of the liver tumors decreased from 8.3 (±4.8) to 1.9 (±1.6) mm. *Significance*. Surf-X-Bio can accurately track liver tumors from combined surface imaging and x-ray imaging. The fast computational speed (<250 milliseconds per inference) allows it to be applied clinically for real-time motion management and adaptive radiotherapy.

## 1. Introduction

The efficacy of radiotherapy relies on the precise delivery of radiation doses to a space highly conformal to the tumor (Verellen *et al* 2007), to ensure patient safety and achieve local disease control (Tubiana and Eschwege 2000). However, patients’ physiological motion, mainly respiratory motion, introduces location uncertainties of tumors for sites like liver (Langen and Jones 2001, van Herk 2004), thereby undermining the accuracy of tumor targeting and increasing the radiation-induced toxicity to the surrounding normal tissues (Bertholet *et al* 2019). Modern radiotherapy linear accelerators (LINACs) are often equipped with x-ray based on-board imaging hardware, which can acquire x-ray images to localize the tumor and monitor its motion before or during the treatment for patient setup correction and motion management (Jaffray 2012, Dhont *et al* 2020). To capture the fast respiratory motion and assess its impact on dose delivery, real-time imaging is highly desired for intra-treatment tumor localization and motion management (Bertholet *et al* 2019, Keall et al 2019). For real-time imaging and its application in delivery adaptation, the overall latency is required to be contained within 500 milliseconds (ms) such that the fast tumor motion can be resolved with simultaneous treatment action/interference (Keall *et al* 2021).

Traditional methods of real-time imaging either directly track tumors from two-dimensional (2D) on-board x-ray projections or fluoroscopic images, or indirectly monitor surrogates such as fiducial markers or anatomic landmarks (Bertholet *et al* 2019). However, these methods have different drawbacks for the site of liver. For example, directly tracking liver tumors on x-ray projections is extremely challenging due to its poor contrast against the surrounding liver parenchyma (Dhont *et al* 2020, Gupta *et al* 2020). Inserting radiopaque fiducial markers into livers as tracking surrogates requires an invasive procedure which can lead to inflammation, and the markers may migrate over time after the insertion (Bhagat *et al* 2010, Roman *et al* 2012). The diaphragm has served as an anatomical surrogate for liver tumors, as the position of the diaphragm can be easily tracked from x-ray projections (Balter *et al* 2001, Hirai *et al* 2020). However, the correlation of motion between the liver tumor and the diaphragm is case-dependent and affected by the intra-liver tumor position (Yang *et al* 2014). Due to the stringent temporal requirements of real-time imaging (<500 ms), all these methods are also based on planar imaging that only captures tumor motion in 2D, while 3D tumor localization is more relevant to the real-time motion management. Compared with 2D planar imaging, 3D imaging captures the 3D motion and deformation of the tumor, thus allowing more precise targeting and simultaneous treatment dose calculation/accumulation (Tubiana and Eschwege 2000, Keall 2004, Shirato *et al* 2004). However, cone-beam computed tomography (CBCT) imaging, the current on-board x-ray volumetric imaging technique, requires substantial scan time on the order of ∼1 min. The achievable temporal resolution by current clinical CBCT imaging systems differs from the real-time temporal requirement by two orders of magnitude. The low x-ray contrast of liver, combined with the real-time temporal restriction, poses substantial challenges to real-time volumetric imaging for 3D liver tumor localization.

To bridge the gap between the hardware constraints and the real-time 3D imaging needs, recently there have been growing interests in applying deep learning (DL)-based approaches to address real-time volumetric imaging and tumor localization problems (Litjens *et al* 2017, Fu *et al* 2020). Various DL architectures and training schemes were proposed to reconstruct 3D volumetric images from a single or two orthogonal-view x-ray projections (Shen et al 2019, Ying *et al* 2019, Lei *et al* 2020). However, these studies are mostly based on the site of lung, which has much higher soft tissue contrast than liver. For these methods, additional steps of image registration or segmentation are required to further localize the liver tumors from the reconstructed images, which can be challenging for the low-contrast liver. Instead of reconstructing real-time CBCT images, Wei *et al* developed a DL network to solve deformation-vector-fields (DVFs) to track lung tumors in 3D from a single x-ray projection, using a principal component analysis (PCA)-based deformation model established from a prior planning 4D-CT set (Wei *et al* 2020). The DVF-driven approach allows simultaneous 3D tumor tracking and volumetric image estimation. However, the PCA-based approach may not be able to resolve motion not contained in the 4D-CT images, particularly the rigid motion. The low soft-tissue contrast of liver may further reduce its accuracy.

Another DVF-driven approach (MeshRegNet-Bio) was developed in a previous pilot study to achieve real-time 3D liver imaging and tumor localization (Shao *et al* 2022). MeshRegNet-Bio addresses the extreme under-sampling challenge of real-time imaging by combining prior information, graph neural network (GNN)-based deformable registration, and biomechanical modeling. Trained by patient-specific 4D-CT images, a GNN-based deformable registration network was capable to deform a prior liver boundary mesh to match image features encoded in a single x-ray projection. The estimated 3D liver boundary motion/deformation was further used as the boundary condition to input into a liver biomechanical model to localize the intra-liver tumors in real-time. Compared with the PCA-based model (Wei *et al* 2020), our approach allows training for a mixture of rigid/deformable motion. It also incorporates the domain knowledge from biomechanical modeling to address the low-contrast liver issue. While the pilot study successfully demonstrated the feasibility, several limitations remain to be addressed: (a) in the pilot simulation study, a wide field-of-view (FOV) was used for the x-ray projection to cover the transverse span of patients’ abdomen, such that the whole liver volume can be included. However, in real clinics such a wide FOV may not be guaranteed. For smaller FOVs, part of the liver mesh under registration may not have corresponding features within the x-ray projection to infer the local liver boundary deformation, which may lead to inaccuracy/instability. (b) The photon and electronic noises were not accounted for in the pilot study. In addition to the primary signal, x-ray cone-beam projection also contains signal-degradation noises (Jia *et al* 2012). Since noises can obscure subtle image features, removing them may lead to overly optimistic results. (c) For the mesh-based deformable registration network, previous studies have found it less robust in scenarios with large motion, as the motion-encoded image features may be further away from the original nodes of the prior liver mesh (Nakao *et al* 2022), and difficult for the network to capture. (d) For biomechanical modeling, a conventional finite-element solver was used to solve the intra-liver deformation, which took ∼30 s and remained the efficiency bottleneck of the overall real-time framework.

To address the above challenges/limitations, in this work another real-time imaging modality was utilized to improve the tumor localization accuracy and robustness, especially for large motion. Besides on-board x-ray imaging, optical surface imaging provides another real-time imaging modality that can monitor a large FOV (up to 110 × 140 × 240 cm^3^) with a sub-millimeter detectability (Meeks *et al* 2005, Hoisak and Pawlicki 2018, Padilla *et al* 2019, Freislederer *et al* 2020, Al-Hallaq *et al* 2022, Li 2022). With the non-ionizing light sources and high frame rates of monitoring systems [10–24 Hz (Al-Hallaq *et al* 2022)], opitcal imaging can continuously monitor patients’ body surface in real-time without incurring additional dose. Recently, surface imaging has also been proposed for real-time adaptive radiotherapy (Batista *et al* 2020, Wang *et al* 2021, Huang *et al* 2022). However, the accuracy of tumor localization relies on the motion correlation between the external body surface and the internal anatomy. As such correlations can be complex and imperfect, on-board x-ray imaging can be further used to correct the residual motion estimation errors. The x-ray imaging captures the internal anatomy, while the surface imaging captures the external body variations which are correlated with the internal motion. Combining the information from both modalities allows us to maximize the usage of available real-time signals during the radiotherapy treatment. The large FOV of surface imaging helps to alleviate the challenge (a) as mentioned above. The additional information from the surface imaging can help to address (b) and (c) as well. In addition to introducing optical surface imaging, a new DL model was developed to propagate the solved liver boundary motion into the liver. The population-based DL network replaces the conventional finite-element solver for biomechanical modeling. U-Net was chosen as the network architecture (Ronneberger *et al* 2015), as it has demonstrated performance in extracting local and global imaging features to make accurate predictions (Balakrishnan *et al* 2019, Hesamian *et al* 2019). Similar to conventional biomechanical modeling, the U-Net takes the solved liver boundary motion as input and outputs 3D intra-liver DVFs to track and locate the tumor. However, the network allows a ‘one-pass’ approach to directly infer the intra-liver DVFs from the boundary condition without time-consuming iterative optimization. The DL network was developed to significantly accelerate the overall framework such that all computations can be executed within hundreds of ms to meet the real-time temporal requirements to address the challenge (d). The overall framework combines optical surface imaging-based liver boundary motion estimation, x-ray imaging-based liver boundary motion fine-tuning, and deep learning-based biomechanical modeling (Surf-X-Bio). Via Surf-X-Bio, the body surface imaging was used to estimate the liver boundary node motion, and an x-ray-based GNN model was further used to correct the residual liver boundary registration errors. Then the fine-tuned liver boundary motion was input into the DL-based biomechanical modeling step to localize the intra-liver tumor in real-time. Below the overall Surf-X-Bio framework was introduced first, followed by detailed descriptions of each of its building blocks.

## 2. Methods and materials

### 2.1. Method overview

**Figure 1.**
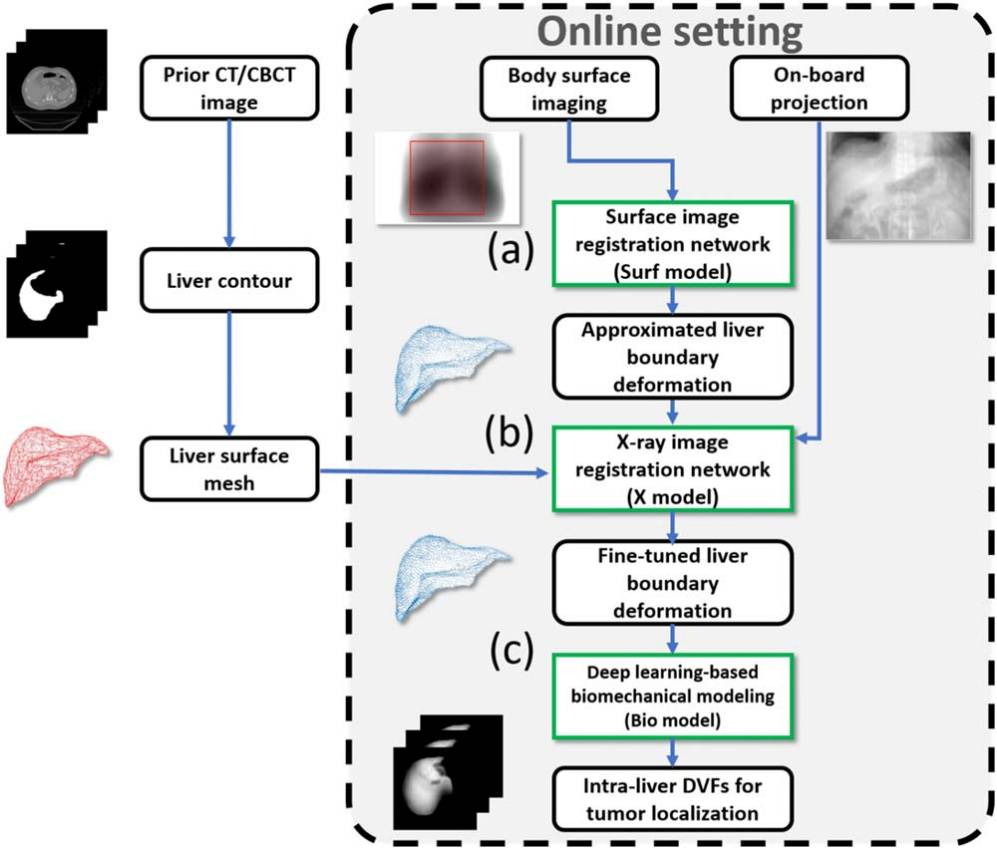
Workflow of the proposed Surf-X-Bio framework to localize liver tumors in real-time, by combing two real-time imaging modalities and a deep learning-driven biomechanical model. In step (a), the Surf model first estimated an initial liver boundary deformation vector field (DVF) from an optical surface image of the thoracic-abdominal region. In step (b), the approximated liver boundary DVFs were further fine-tuned by a graph neural network using the image features encoded in an on-board x-ray projection. Finally, in step (c) a deep learning-based biomechanical model solved the intra-liver DVFs for liver tumor localization, using the liver boundary DVFs from the steps (a) and (b) as the boundary condition. Adapted from Shao *et al* (2022). © 2022 Institute of Physics and Engineering in Medicine. All rights reserved.

As mentioned, Surf-X-Bio localizes the liver tumor volumetrically in real-time via a DVF-driven approach. The deformable registration can be divided into two phases (figure 1). The 1st phase, comprising steps (a) and (b) in figure 1, deforms a prior (reference) liver boundary mesh according to motion features encoded in an optical surface image and a single x-ray projection. Specifically, in step (a), a patient-specific DL model (Surf) was applied to make an initial inference of liver boundary DVFs, using an optical surface image acquired around the thoracic-abdominal region. The Surf model uses the learnt correlations between the respiratory-induced external surface motion and internal liver motion to predict the liver boundary motion. In step (b), using learnt features from x-ray imaging, the residual liver boundary registration errors were further corrected by a GNN-based model (X). The X model uses the patient-specific internal motion features directly captured by the on-board x-ray projection, to fine-tune the DVFs solved by the Surf model. For steps (a) and (b), the prior (reference) liver boundary mesh was generated offline from liver segmentations manually contoured on a prior CT/CBCT image. Representing the liver by mesh allows a flexible description of its complex geometry. The mesh representation also allows us to construct a one-to-one correspondence between the prior and on-board liver mesh nodes. Thus, the liver boundary motion can be explicitly defined and solved for the DVF-driven framework.

In the 2nd phase (step (c) in figure 1), the solved liver boundary motion was input into a DL-based biomechanical model (Bio) to derive the intra-liver DVFs. The organic combination of Surf, X, and Bio models allows Surf-X-Bio to accurately capture the liver and tumor motion at a high frequency (4–5 Hz) to guide real-time radiotherapy. In the following subsections, we detailed the network architecture and training schemes of the Surf-X-Bio framework, followed by the data curation and augmentation procedures, and the evaluation methods/criteria for the liver boundary registration and tumor localization accuracy.

### 2.2. Surface image registration network (Surf model)

The Surf model extracts motion-related features from the optical surface image to predict the liver boundary motion. We built the Surf model as a deep learning model, since deep learning has demonstrated its capability in capturing subtle image features and resolving complex relationships between the extracted features and the desired outputs. Since the motion correlation is highly patient-dependent, we developed Surf as a patient-specific model to learn these patient-specific traits.

**Figure 2.**
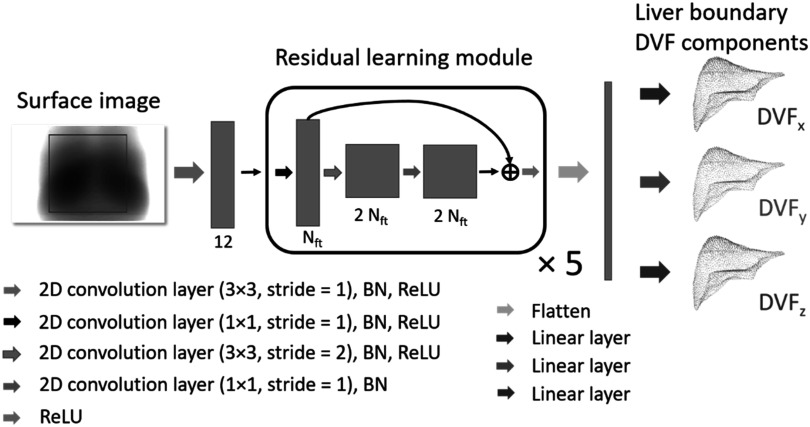
Network architecture of the Surf model. The Surf model predicts liver boundary DVFs from a surface image of the thoracic-abdominal region. The network contains an entrance 2D convolution layer followed by five residual learning modules and three parallel linear layers. The numbers below the blue blocks indicate the numbers of feature maps: for the entrance layer the feature map number was 12, and the number (${N}_{{ft}}$) was doubled in each of the following residual modules. The three linear layers output the three Cartesian components of the liver boundary DVFs, defined at the liver boundary nodes. BN: batch normalization. ReLU: rectified linear unit. DVF: deformation vector field.

The Surf model architecture is illustrated in figure 2. The network inputs were the surface images. The pixel intensity of the surface image represented the vertical coordinates (i.e. the anterior–posterior positions) of the body surface with respect to an arbitrary reference plane. In our application, we defined the image iso-center plane as the reference plane. The network design was adapted from the ResNet architecture (He *et al* 2016) that consists of a series of 2D convolution layers, connected via batch normalization operations and activation functions (ReLU), and followed by three parallel linear layers yielding liver boundary DVFs along the three Cartesian directions. Except for the entrance convolution layer, the rest convolution layers were organized in the residual learning architecture (He *et al* 2016). In total, five residual learning modules were stacked in series. The number of feature maps of the entrance convolution layer was 12, and the number was doubled in each residual learning module. Meanwhile, the image size of the feature maps along each dimension was halved by the second convolution layer in each residual learning module with a stride of two. After the last residual learning module, the feature maps were flattened as a 1D tensor that was further processed by three linear layers, each of which predicted a Cartesian component of the DVFs at the liver surface nodes. The model training was driven by a similarity loss quantified by the mean squared error between the predicted and ‘ground-truth’ liver boundary DVFs:

\begin{eqnarray*}{L}_{{surf}}=\frac{1}{N}\displaystyle \sum _{p=1}^{N}{\parallel {{\boldsymbol{d}}}_{p}-{{\boldsymbol{d}}}_{p}^{{gt}}\parallel }^{2},\end{eqnarray*}

where *N* denotes the number of liver surface nodes, ${{\boldsymbol{d}}}_{p}$ and ${{\boldsymbol{d}}}_{p}^{{gt}}$ denotes the predicted and ‘ground-truth’ liver surface DVFs at the *p*th node, respectively.

### 2.3. X-ray image registration network (X model)

**Figure 3.**
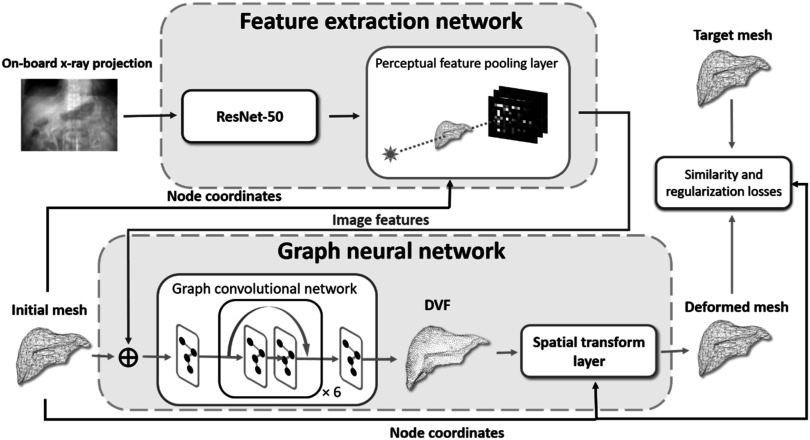
Network architecture of the X model. It consists of a feature extraction subnetwork and a graph neural network. The feature extraction subnetwork extracts image features from an on-board x-ray projection and performs node-based perceptual feature pooling. The pooled features are then fed into the graph neural network for liver boundary DVF prediction. The initial liver boundary mesh for the X model has been deformed by the liver boundary DVFs estimated from the Surf model (section 2.2). DVF: deformation vector field. Adapted from Shao *et al* (2022). © 2022 Institute of Physics and Engineering in Medicine. All rights reserved.

In contrast to the Surf model, the X model extracts image features directly from an on-board x-ray projection to predict liver boundary DVFs. It fine-tunes the estimated liver boundary motion from the Surf model to match the liver boundary mesh with features shown in the on-board x-ray projection. Figure 3 presents the X model architecture adapted from the Pixel2Mesh library (Wang *et al* 2018). It consists of two subnetworks, performing feature extraction and liver boundary motion prediction, respectively. The first subnetwork extracts image features from an x-ray projection, and a following perceptual feature pooling layer that performs liver boundary node-based feature pooling. Due to potentially limited FOVs, the on-board projection may not cover the whole liver. For those projected boundary nodes that are outside of the FOV, their feature values are defaulted to 0. The pooled features via the feature pooling layer are subsequently fed into the graph neural network for liver boundary DVF estimation. The graph neural network consists of a graph convolutional network (GCN) and a spatial transform layer. The input of the GCN includes the initial mesh coordinates and the pooled features. The GCN predicts the liver boundary DVFs, which is used in the subsequent spatial transform layer to deform the initial liver boundary mesh to its final position/shape.

The loss function for the X model involves a similarity loss, a Laplacian loss, and a deformation energy loss. Since there is a one-to-one correspondence between the nodes of the predicted and ‘ground-truth’ liver boundary meshes, the similarity loss ${L}_{{sim}}$ is defined as direction-weighted mean squared errors between the two surface meshes:

\begin{eqnarray*}{L}_{{sim}}=\frac{1}{N}\displaystyle \sum _{p=1}^{N}\left({({x}_{{LR},p}-{x}_{{LR},p}^{{gt}})}^{2}+{({x}_{{AP},p}-{x}_{{AP},p}^{{gt}})}^{2}+{{a}_{{SI}}({x}_{{SI},p}-{x}_{{SI},p}^{{gt}})}^{2}\right),\end{eqnarray*}

where ${a}_{{SI}}$ denotes the weighting factor for the superior–inferior (SI) direction, ${x}_{{LR},p},$ ${x}_{{AP},p},$ and ${x}_{{SI},p}$ respectively denote the coordinates of the *p*th deformed nodes along the left–right (LR), anterior–posterior (AP), and SI directions. We used weighting factors of 4 for ${a}_{{SI}},$ since the SI direction is the most prominent direction for the respiratory motion and was assigned a larger weighting. The Laplacian loss (${L}_{{lap}}$) is used to regularize the boundary DVFs to prevent un-realistic large local deformations (Wang *et al* 2018). It calculates the mean squared distances between the Laplacian coordinates before (${{\boldsymbol{\delta }}}_{p}$) and after (${{\boldsymbol{\delta }}}_{p}^{{\prime} }$) a deformation:

\begin{eqnarray*}{L}_{{lap}}=\frac{1}{N}\displaystyle \sum _{p}{\parallel {{\boldsymbol{\delta }}}_{p}^{{\prime} }-{{\boldsymbol{\delta }}}_{p}\parallel }^{2},\end{eqnarray*}

where the Laplacian coordinates are defined by

\begin{eqnarray*}{{\boldsymbol{\delta }}}_{p}={{\boldsymbol{x}}}_{p}-\displaystyle \sum _{q\in {\mathscr{N}}\left(p\right)}\frac{{{\boldsymbol{x}}}_{q}}{\left|{\mathscr{N}}\left(p\right)\right|}.\end{eqnarray*}

Here, ${\mathscr{N}}\left(p\right)$ denotes the neighboring nodes of the *p*th node. The 3rd loss function term, deformation energy, measures the mean deformation energy of the predicted DVFs:

\begin{eqnarray*}{L}_{{eng}}=\frac{1}{N}\displaystyle \sum _{p}{{\boldsymbol{d}}}_{p}\bullet \left({{\boldsymbol{d}}}_{p}-\displaystyle \sum _{q\in {\mathscr{N}}\left(p\right)}\frac{{{\boldsymbol{d}}}_{q}}{\left|{\mathscr{N}}\left(p\right)\right|}\right),\end{eqnarray*}

where ${{\boldsymbol{d}}}_{p}$ denotes the liver surface DVFs at the *p*th node. The total loss function is a weighted sum of the above similarity and regularization losses:

\begin{eqnarray*}{L}_{{tot}}={\lambda }_{{sim}}\,{L}_{{sim}}+{\lambda }_{{lap}}\,{L}_{{lap}}+{\lambda }_{{eng}}\,{L}_{{eng}},\end{eqnarray*}

where λ defines the weighting coefficient. We used ${\lambda }_{{sim}}=1,$ ${\lambda }_{{lap}}=0.05,$ and ${\lambda }_{{eng}}=1$ after trial-and-error based optimizations.

### 2.4. Deep learning-based biomechanical modeling (Bio Model)

**Figure 4.**
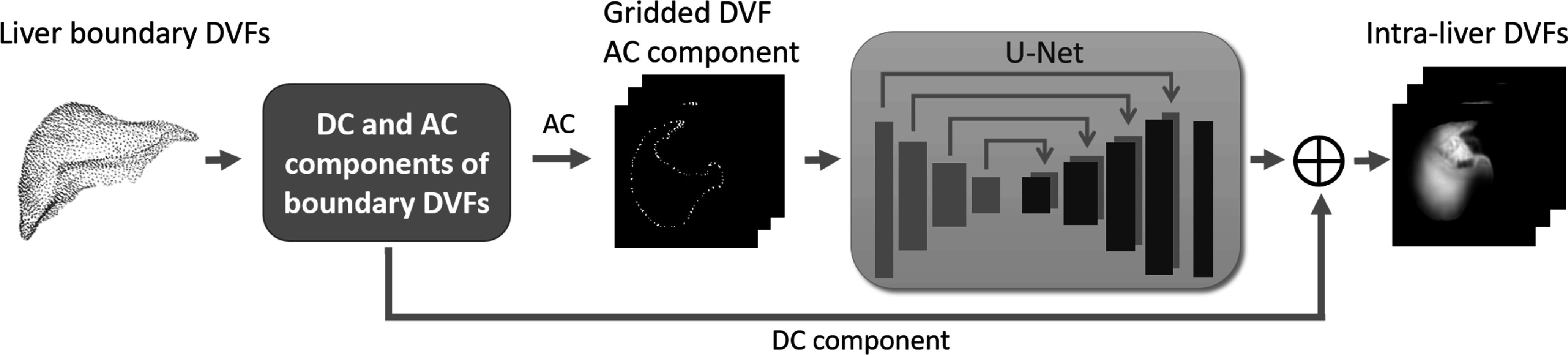
Workflow of the Bio model. The liver boundary DVFs defined on a boundary mesh are first decomposed into a spatially uniform (DC) component and an oscillating (AC) component, respectively. The AC component is gridded into a Euclidean volumetric grid before input into the U-Net. Afterwards, the DC component of the DVFs (representing rigid motion) is added back to the U-Net prediction (representing intra-liver deformable motion) to yield the intra-liver DVFs. DVF: deformation vector field.

The workflow of the Bio model is illustrated in figure 4. The Bio model was based on the U-Net architecture (Ronneberger *et al* 2015). To facilitate feature learning, the liver boundary DVFs solved by the preceding steps are decomposed into a spatially uniform component and a residual oscillating component (respectively called the DC and AC components in figure 4). Since the uniform DC component represents a rigid motion, only the AC component is inputted into the U-Net to estimate intra-liver deformation. The DC component is subsequently added back to the U-Net predictions to yield the overall intra-liver DVFs. Since the U-Net requires regular Euclidean data inputs, before fed into the U-Net, the mesh-based AC component is gridded into a Euclidean volumetric grid via nearest neighboring. The liver biomechanical modeling was based on a Mooney–Rivlin material model (Zhang *et al* 2019, Shao *et al* 2021) that generates the ground-truth intra-liver DVFs for model training.

The U-Net architecture consists of a contraction and an expansion path linked by skip connections. The contraction path contains five blocks, and each block includes a 3D convolution layer with a stride of 2 and a leaky ReLU activation function with a negative slope of 0.2. All 3D convolution layers in the U-Net have a kernel of size 3 × 3 × 3. Prior to the contraction path, we added a block containing a 3D convolution layer with a stride of 1 and a leaky ReLU activation function. The feature number of the first convolution layer was 8, and the number was doubled after each 3D convolution in the contraction path. The expansion path, symmetric to the contraction path, also contains five blocks. Each block involves an up-sampling layer, a 3D convolution layer with a stride of 1, and a leaky ReLU activation function. Before the convolution operations in the expansion path, the feature maps are concatenated with the counterparts in the contraction path through the skip connections. The numbers of feature maps are halved after each convolution in the expansion path. The contraction and expansion paths are connected by a 3D convolution layer with a stride of 1. Lastly, a 3D convolution layer with stride of 1 converts the output feature maps of the expansion path to a three-component intra-liver DVFs, each defined for one Cartesian direction. The loss function for the Bio model involves a weighted mean squared error loss between the predicted and ‘ground-truth’ volumetric DVFs. Since we were only interested in the intra-liver DVFs for liver tumor localization, the volumetric DVFs outside the liver were irrelevant. Therefore, the whole DVF volume was divided into a region containing the liver and the outside region, and the similarity losses in the two regions were weighted differently to compute the total similarity loss. The region containing the liver was defined by the dilated prior liver contour with a 1 cm margin. We used the weighting factors 1.0 and 0.01 for the regions including and excluding the liver, respectively. The small loss applied to the latter serves to prevent the network from making arbitrary inferences there.

### 2.5. Dataset curation, augmentation, and model training

A dataset of 34 patients with liver cancer was retrospectively collected from our institute to train and evaluate the proposed Surf-X-Bio framework. Each patient had a contrast-enhanced 4D-CT scan with liver tumors contoured by experienced physicians. The details of the dataset have been presented previously (Shao *et al* 2021), thus only some key characteristics were summarized in table 1.

**Table 1.** Characteristics of the 4D-CT dataset. The phase 0% is defined at the end-of-inhale phase.

Number of respiratory phases	Respiratory phases	Number of patients	Volume size	Resolution (mm^3^)
2	0%, 50%	1	256 × 256 × 128	2 × 2 × 2
3	0%, 50%, 90%	7	256 × 256 × 128	2 × 2 × 2
10	0%–90%	26	256 × 256 × 128	2 × 2 × 2

As the Surf-X-Bio framework involves multiple DL networks that take different input/output data structures and were trained separately, the strategies of data augmentation and model training were tailored for each of them. For Surf-X-Bio, we trained the Surf and X as patient-specific models to capture patient-dependent motion features. For the Bio model, we trained it as a population-based model since the finite-element solver can be considered as a population-based solver. The patient dataset was correspondingly partitioned into two subsets for the patient-specific models and the population-based model. The 1st subset contained 10 patients randomly selected from the cases with 10-phase 4D-CT scans, and the remaining patients (24) in the dataset were assigned to the 2nd subset. The 1st dataset was used to train/validate/test the patient-specific Surf and X models, as well as testing the population-based Bio model. The 2nd subset was used exclusively to train/validate the population-based Bio model. This partition strategy guarantees that the Bio model had never seen any information from the 1st subset during the training stage.

Since each patient in the 1st subset had only a 10-phase 4D-CT which was insufficient to train the patients-specific Surf model, we augmented each 4D-CT by simulating more variations of respiratory motions, via a PCA-based model (Shao *et al* 2022). The details of the deformable augmentation are available in our pilot study, thus only the key information was summarized here: (1) for each patient, we performed deformable registrations between the 0% 4D-CT phase (used as prior) and the other phases (10%–90%), using the open-source toolbox Elastix (Klein *et al* 2010). For each CT image, we contoured and density-overrode the liver volume to enhance its boundary contrast and improve the registration accuracy there; (2) we extracted the DVFs at the liver boundary nodes to feed into a finite-element solver via the open-source FEBio package (Maas *et al* 2012), to generate biomechanical modeling-enhanced intra-liver DVFs; (3) a patient-specific motion model was created by performing PCA on the resulting DVFs. The PCA formulates a patient’s DVF at a respiratory phase *t*% as a linear combination of the mean DVF (${{\boldsymbol{D}}}_{0}$) and principal motion components (${{\boldsymbol{D}}}_{i}(i=1-3)$):

\begin{eqnarray*}{{\boldsymbol{D}}}^{t \% }\left({\boldsymbol{x}}\right)=\displaystyle \sum _{i=0}^{3}{w}_{i}^{t \% }\,{{\boldsymbol{D}}}_{i}\left({\boldsymbol{x}}\right),\end{eqnarray*}

where ***x*** denotes the voxel index, and ${w}_{i}^{t \% }$ denotes the PCA coefficient of ${{\boldsymbol{D}}}_{i}$ at the phase *t*%. Only the first three motion components were used in equation (7), as they were demonstrated sufficient in describing respiratory motion (Li *et al* 2011); (4) finally, we augmented the deformable motions by randomly scaling the coefficients ${w}_{i}^{t \% }$ of the mean and principal motion components, similar to Jiang *et al* (2021). The scaling range for ${w}_{0}^{t \% }$ was [0.95, 1.05], and the ranges for the 1st, 2nd, and 3rd coefficients were [−1.5, 3.0], [−1.5, 3.0], and [−1.5, 1.5], respectively. For each phase *t*%, we independently and respectively sampled 4, 6, 4, and 2 scaling factors from these intervals with uniform density probability. We applied the augmentation strategy to phases 10%–40% of the original 4D-CT scan to generate 768 cases for training, and to phases 70%–80% to generate 384 cases for validation. In addition to the deformable augmentation, rigid translations were performed on-the-fly along the AP, LR, and SI directions during the network training. The translation was independently sampled for each direction at a uniform density probability, within a range of [−6 mm, 6 mm].

For each 4D-CT, the 50%, 60%, and 90% phases were left out for testing. This phase partitioning was adopted to render the testing phases closer to the extrema of the respiratory cycle (i.e. close to the end-of-exhale and end-of-inhale). Accordingly, the testing sets had larger intra-set motion, and also larger variation/deformation to the training/validation cases. For the testing set, we simulated four types of motion scenarios deviating from the training and validation sets to avoid potential data leakage and also to test the model robustness. The testing cases were generated by scaling the PCA coefficients ${w}_{i}^{t \% }$ of the 50%, 60%, and 90% phases with scaling factors deviating away from those used in the training and validation sets, or by applying a larger translation. The first motion type simulates varying breathing amplitudes, by which the scaling factors for ${w}_{1}^{t \% }$ and ${w}_{2}^{t \% }$ of the testing phases were sampled in the range of [0.0, 5.0] (compared to [−1.5, 3] of training/validation), and the scaling factor for ${w}_{3}^{t \% }$ was sampled in the range of [0.0, 2.5] (compared to [−1.5, 1.5] of training/validation). Twenty-five scaling factors were sampled from these intervals for each phase, resulting in 45 testing cases for each patient. The remaining three types of motion scenarios involve both the respiratory and translational motions. In these scenarios, the scaling factors of the PCA coefficients were kept as unity (i.e. corresponding to the original testing phases), while the range of the translational motion was gradually varied from −10 to 10 mm in the LR (scenario type 2), AP (scenario type 3), and SI (scenario type 4) directions, respectively. For each testing phase, we sampled 11 cases with uniform spacing along each direction. In total, 33 testing cases were simulated of each patient for each motion scenario (2, 3, or 4). Note the scanning range ([−10 mm, 10 mm]) was beyond the range ([−6 mm, 6 mm]) used to generate the training and validation cases.

After curating all samples, we simulated the optical surface images from the augmented CT images. We first constructed surface triangular meshes from body contours of the CT images. The maximum radius of the Delaunay sphere was chosen to be 2.5 mm. Then the vertical coordinates (i.e. the AP reading of the body surface mesh nodes relative to the iso-center plane) were resampled into a Euclidean grid using linear interpolation to represent the surface image. In our study, the surface image captures a rectangle region with 260 × 260 pixels, spanning a FOV of 202 × 202 mm^2^ (Al-Hallaq *et al* 2022). To simulate imperfect internal–external motion correlations, systematic and random noises were added on-the-fly to the surface map during the training stage to reduce the correlation. Three types of noises were added to the surface image: (1) rigid, random translational uncertainty within ±40% of the mean motion magnitude. (2) Random deformable uncertainty in forms of the Gaussian noise. The standard deviation of the noise was chosen as 100% of the pixel-wise surface deformable motion (to the prior surface) or bounded at 5 mm if the amplitude of the surface motion was too small. (3) Internal–external phase mismatch. Specifically, we simulated a 50% chance that the phase of the target liver boundary DVF could deviate from the phase of the surface motion by 10%.

To train the X model, we also simulated on-board x-ray projections from the augmented 4D-CT images. The size of the projections was 512 × 384 with a resolution of 0.776 × 0.776 mm^2^. The cone-beam projections were generated using a ray-tracing algorithm (Han *et al* 1999), and photon noises were added to the projections. The noise model was a combination of the Poisson and Gaussian noises which simulated the quantum noises of x-ray photons and electronic noises of the detector, respectively (Wang and Gu 2013, Zhang *et al* 2013). For each projection pixel, the mean quantity of x-ray photon was set to 10^5^ for the Poisson noise, and the standard deviation was set to 10 photons for the electronic Gaussian noise. The photon noises were added on-the-fly during the training stage. Examples of the simulated surface images and on-board x-ray projections were provided in Supplementary materials. As described in section 2.3, the X model uses the Surf model’s prediction as the initial input, to further fine-tune the liver boundary DVFs via information extracted from the x-ray projection.

The Surf model was implemented and trained using the PyTorch 1.9.0 library (Paszke *et al* 2019). A learning rate scheduler was used to reduce the learning rate by 40% if the validation loss stopped decreasing for more than 10 epochs. The starting learning rate was 2 × 10^−4^. We used the Adam optimizer with a weight decay of 1 × 10^−7^. The total number of epochs was 200, and the best model in terms of the validation loss was selected for testing. Similar to the Surf model, the X model was also trained using PyTorch, using the predictions from the trained Surf model as input. A learning rate scheduler was set to decrease the learning rate by 40% for every 30 epochs. The initial learning rate was 2 × 10^−4^. The total number of epochs was set to 250, and the best model in terms of the validation loss was selected for testing.

Unlike the Surf and X models, the Bio model was a population-based DL model. It was thus trained/validated and tested on different patients. In detail, we used the 2nd subset of patients, composed of 24 cases to train and validate the Bio model, which was further tested on the same 10 cases as the Surf and X models (1st subset). The 2nd subset underwent similar augmentations as performed for the 1st subset to better train/validate the Bio model. For the Bio model, a learning rate scheduler was used to reduce the learning rate by 10% if the validation loss stopped decreasing for more than 10 epochs. The initial learning rate was 2 × 10^−4^. Adam optimizer was used without weight decay. The total number of epochs was set to 800 and the best model in terms of the validation loss was chosen for testing.

### 2.6. Evaluation and ablative studies

We evaluated both the registration accuracy of the liver boundary mesh and the localization accuracy of liver tumors by the Surf-X-Bio framework. The liver boundary registration accuracy was assessed using the root mean squared error (RMSE) and 95-percentile Hausdorff distance (HD95). Specifically, RMSE quantifies the node-wise distances between the predicted and ‘ground-truth’ liver boundary meshes:

\begin{eqnarray*}\mathrm{RMSE}=\sqrt{\frac{1}{N}\displaystyle \sum _{p=1}^{N}\,{\parallel {{\boldsymbol{x}}}_{p}-{{\boldsymbol{x}}}_{p}^{{gt}}\parallel }^{2}},\end{eqnarray*}

where *N* denotes the number of liver boundary nodes, and ${{\boldsymbol{x}}}_{p}$ and ${{\boldsymbol{x}}}_{p}^{{gt}}$ denote the predicted and ‘ground-truth’ target node coordinates, respectively. The HD95 between two surface meshes *X* and *Y* are defined as

\begin{eqnarray*}\mathrm{HD}95={\mathrm{\max }}\left\{{P}_{95}\,d\left({\boldsymbol{x}},Y\right),{\,P}_{95}\,d\left({\boldsymbol{y}},X\right)\,\right\},\end{eqnarray*}

where ${P}_{95}$ is the 95-percentile operator, and

\begin{eqnarray*}d\left({\boldsymbol{x}},Y\right)=\mathop{{\mathrm{\min }}}\limits_{{\boldsymbol{y}}\in Y}\parallel {\boldsymbol{x}}-{\boldsymbol{y}}\parallel \end{eqnarray*}

is the surface distance between a node ***x*** in *X* and the surface mesh *Y*.

The liver tumor localization accuracy was evaluated using contour-based metrics including the center-of-mass error (COME), the Dice similarity coefficient (DSC), and the HD95. The COME measures the distance between the mass-centers of the predicted and ‘ground-truth’ liver tumor contours. Given two liver tumor contours *V*_1_ and *V*_2_ represented as volumetric binary masks, the DSC is defined by

\begin{eqnarray*}{\mathrm{DSC}}=\displaystyle \frac{2\times \left|{V}_{1}\cap {V}_{2}\right|}{\left|{V}_{1}\right|+\left|{V}_{2}\right|},\end{eqnarray*}

where $\left|V\right|$ denotes the cardinality of *V*. DSC measures the relative overlap between the deformed and ‘ground-truth’ tumor contours. The tumor HD95 is similarly defined as the liver HD95. The ‘ground-truth’ liver and tumor contours were generated by propagating the liver and tumor contours manually segmented from the prior CT images (0%) to the testing motion scenarios (via the augmented motion fields as described in section 2.5). The registration accuracy was evaluated at 0° and 90° projection angles to study the relations between the projection angle and the model performance, as the X model was trained for a specific projection angle. The 0° corresponds to the AP view, and 90° corresponds to the left-lateral view.

Ablative studies were performed to assess the advantages of combining both surface imaging and on-board x-ray projection for the Surf-X-Bio framework. First, the Surf or the X model was ablated from the combined model (Surf-X-Bio) to each individual component, which was compared with the combined Surf-X-Bio model in terms of the liver boundary registration accuracy. The individual X model used for the ablative study was retrained using the prior liver mesh and zero liver boundary DVFs as input. The Surf and X models were also concatenated with the Bio model, respectively called the Surf-Bio and X-Bio models in this study, to perform the DL-based biomechanical modeling to assess their liver tumor localization accuracy and compare with the Surf-X-Bio framework.

The statistical significance of the registration result differences between different models was estimated by the Wilcoxon matched-pairs signed-rank test for the following reasons. First, the underlying statistical distribution is unknown and may not be normally distributed. Moreover, the registration/localization errors of the models were matched and paired. Thus, the nonparametric Wilcoxon matched-pairs signed-rank test was employed (Corder and Foreman 2014). In addition to the calculation of *p*-values, median differences and 95% confidence intervals were estimated by the Hodge–Lehmann estimator (Corder and Foreman 2014).

### 2.7. Network hyperparameter optimization

As the Surf, X, and Bio models contain a number of hyperparameters that can affect the model performance, network hyperparameter tunings were separately performed on each model. For the Surf and Bio models, various combinations of network architecture depths and feature map numbers were evaluated for their effects on the model performance. The model performance was evaluated by the liver surface and liver tumor registration accuracy for the Surf and Bio models, and significance of the difference was evaluated by the Wilcoxon matched-pairs signed-rank test. The optimal Surf model was selected based on the lowest liver surface RMSE, and the optimal Bio model was selected according to liver tumor COME and DSC. Relevant details are provided in Supplementary materials.

For the X-model, which was adapted from our previous work (Shao *et al* 2022), an optimization study has already been performed to finalize the best backbone network (ResNet-50, please see Supplementary file of Shao *et al* 2022). In the present study, we introduced a weighting factor ${a}_{{SI}}$ [equation (2)] to the liver surface mesh similarity loss, to enhance the liver surface registration accuracy in the motion-dominant SI direction. The weighting factor ${a}_{{SI}}$ remains to be optimized. Thus, a comprehensive evaluation of the weighting factor ${a}_{SI}$ was performed, with values ranging from 1 to 5. It was found that ${a}_{{SI}}=4$ provided the best results for the 0° projection, based on the liver boundary RMSE. Accordingly, weighting factor ${a}_{{SI}}=4$ was chosen to train the X-Bio and Surf-X-Bio models. Relevant details are provided in Supplementary materials.

### 2.8. Robustness test to iso-center miscalibration between surface and x-ray imaging

As the proposed framework utilized two imaging modalities to estimate liver surface deformation, we implicitly assumed that the two imaging devices were well calibrated and shared a universal coordinate system. However, in practice miscalibrations between the two imaging devices can exist. While random noises were added during the training stage of the Surf-X-Bio model to enhance the model robustness, it remains unclear the capability of the Surf-X-Bio model to correct the potential miscalibration between the two devices (systematic offset). Thus, a robustness test was performed on the Surf-X-Bio model to assess its response to calibration errors. For each testing case, a relative shift was added to the iso-centers of the two imaging devices. The shift was simulated as a rigid translation and added to the DVFs predicted by the Surf model before inputting into the following X model. The magnitude of the shift was fixed, but the orientation of the shift was randomized in 3D for each testing case. We used amplitudes of 1.0 and 2.0 mm, and the corresponding liver boundary registration and tumor localization errors were evaluated to assess the model robustness.

## 3. Results

### 3.1. Liver boundary registration accuracy

**Figure 5.**
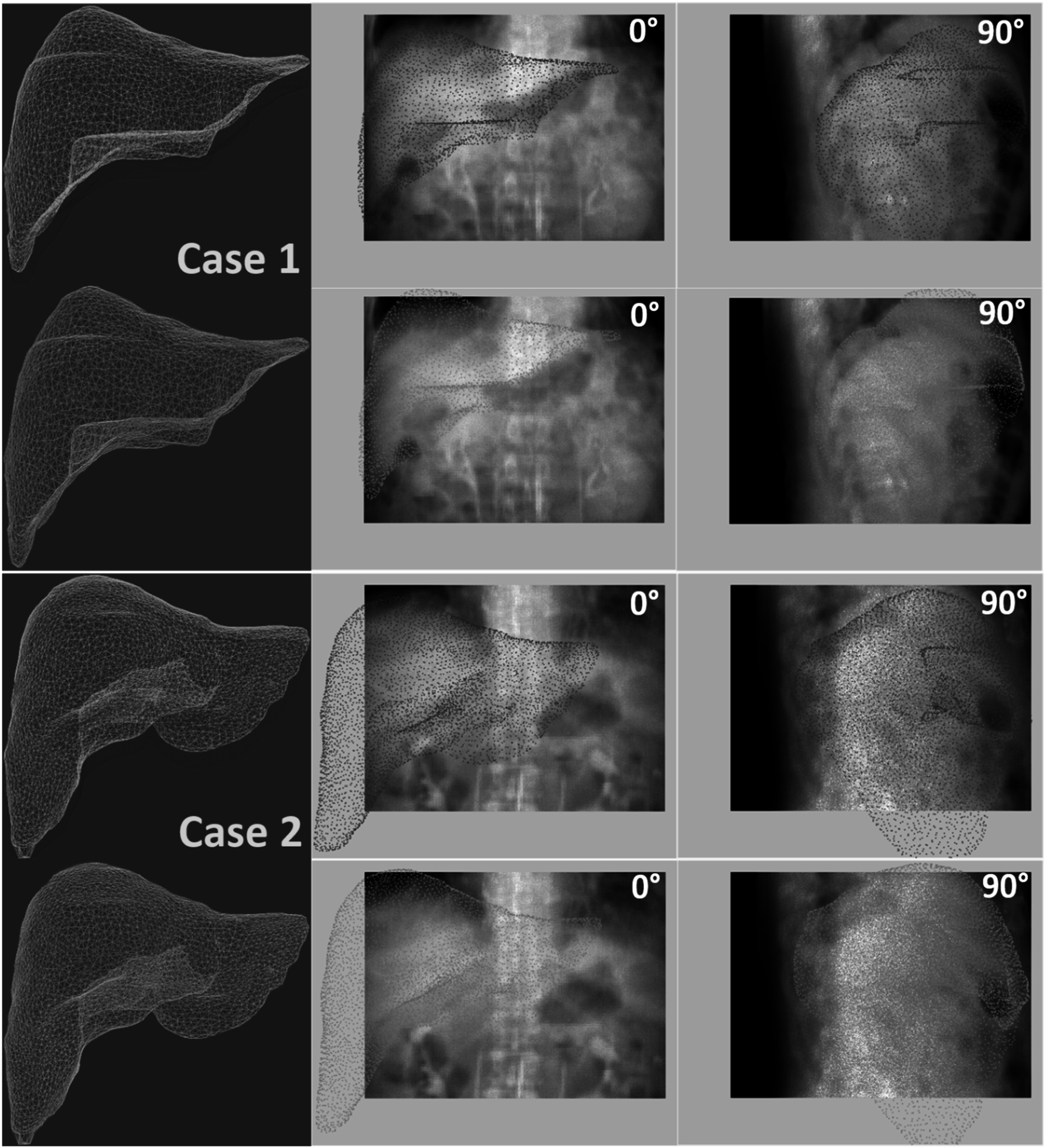
Liver boundary registration results of Surf-X-Bio for two patient cases. The 1st column shows the mesh overlays between the pre-registration (upper panel) and post-registration (lower panel) liver boundary meshes of each patient case. The ‘ground-truth’ target mesh is shown in yellow, and the prior and predicted meshes are shown in red and green, respectively. The other columns project the pre-registration and post-registration liver boundary nodes onto the x-ray projections at two projection angles (0° and 90°). The red and green dots represent the projected nodes of the prior and deformed liver boundary meshes, respectively.

Figure 5 presents a qualitative comparison between pre- and post-registration liver boundary meshes for two patient cases at 0° and 90° projection angles, respectively. Both cases were selected from the motion scenario type 4, combining the end-of-exhale motion (50%) with a 5 mm translation along the superior direction. The 1st column shows mesh overlays between the prior and ‘ground-truth’ target meshes (upper panel) and between the predicted and target meshes (lower panel). The upper panel of each patient reflects the degree of the on-board motion, and the lower panel shows the registration accuracy. The other columns of figure 5 show overlays of the pre- and post-registration surface mesh nodes projected onto the corresponding on-board x-ray images at the two projection angles. The second patient case shows the FOV of the projection fails to cover all the liver mesh, especially for the 0° projection. However, even with such a limited FOV, the Surf-X-Bio model can estimate accurate liver boundary motion.

Due to the limited space, a detailed comparison of the liver registration accuracy of the patients and motion scenarios were provided in Supplementary materials. Here, in table 2 we summarized the quantitative results of the liver boundary registration accuracy and the estimation of the median difference and 95% confidence interval between the Surf-Bio and Surf-X-Bio models and between the X-Bio and Surf-X-Bio models. For naming consistency, the suffix -Bio was added to the model names, but the Bio model was not yet implemented for the liver boundary results. All *p*-values of the Wilcoxon signed-rank tests between the Surf-Bio, X-Bio, and Surf-X-Bio models reached the significance level (*p* < 0.05). Table 2 shows that the Surf-X-Bio model reduced the residual registration errors from the Surf-Bio model and outperformed the X-Bio model. It can also be observed that the means between the X-Bio model and the Surf-X-Bio model had larger differences than those of the medians, which was contributed by the relatively poor performance of the X-Bio model in scenarios of large motion.

**Table 2.** Liver boundary registration results (RMSE and HD95) of the Surf-Bio, X-Bio, and Surf-X-Bio models for all tested motion scenarios. The projection angle denotes the scan direction of the on-board x-ray projections. The RMSE and HD95 are presented in terms of means and standard deviations [$\bar{X}\pm {\mathrm{SD}}$], and medians and quartiles [*M* (*Q*_1_, *Q*_3_)]. The last two columns calculate the median differences (95% confidence intervals) between the Surf-Bio and Surf-X-Bio models and between the X-Bio and Surf-X-Bio models, calculated using the Hodge–Lehmann estimator (Corder and Foreman 2014).

			Model			Median difference	
Metric	Projection angle	Prior	Surf-Bio	X-Bio	Surf-X-Bio	Surf-Bio/Surf-X-Bio	X-Bio/Surf-X-Bio
RMSE (mm)	0°	7.8 ± 3.5	2.0 ± 1.6	2.7 ± 2.9	**1.5 ± 1.3**
				1.4 (1.0, 3.0)	**1.0 (0.7, 1.8)**	0.4 (0.3, 0.4)	0.4 (0.4, 0.5)
	90°	7.8 (5.4, 10.0)	1.5 (0.9, 2.6)	2.5 ± 2.7	**1.6 ± 1.3**
				1.3 (0.9, 2.6)	**1.1 (0.8, 1.8)**	0.3 (0.3, 0.3)	0.3 (0.3, 0.4)
HD95 (mm)	0°	9.8 ± 4.5	2.9 ± 1.9	3.8 ± 3.1	**2.3 ± 1.6**
				2.5 (1.8, 4.4)	**1.7 (1.2, 2.8)**	0.5 (0.5, 0.6)	0.8 (0.8, 0.9)
	90°	9.6 (6.6, 12.6)	2.5 (1.6, 3.7)	3.5 ± 2.9	**2.4 ± 1.6**
				2.4 (1.7, 3.9)	**1.8 (1.3, 2.9)**	0.4 (0.4, 0.5)	0.7 (0.6, 0.7)

### 3.2. Liver tumor localization accuracy

**Figure 6.**
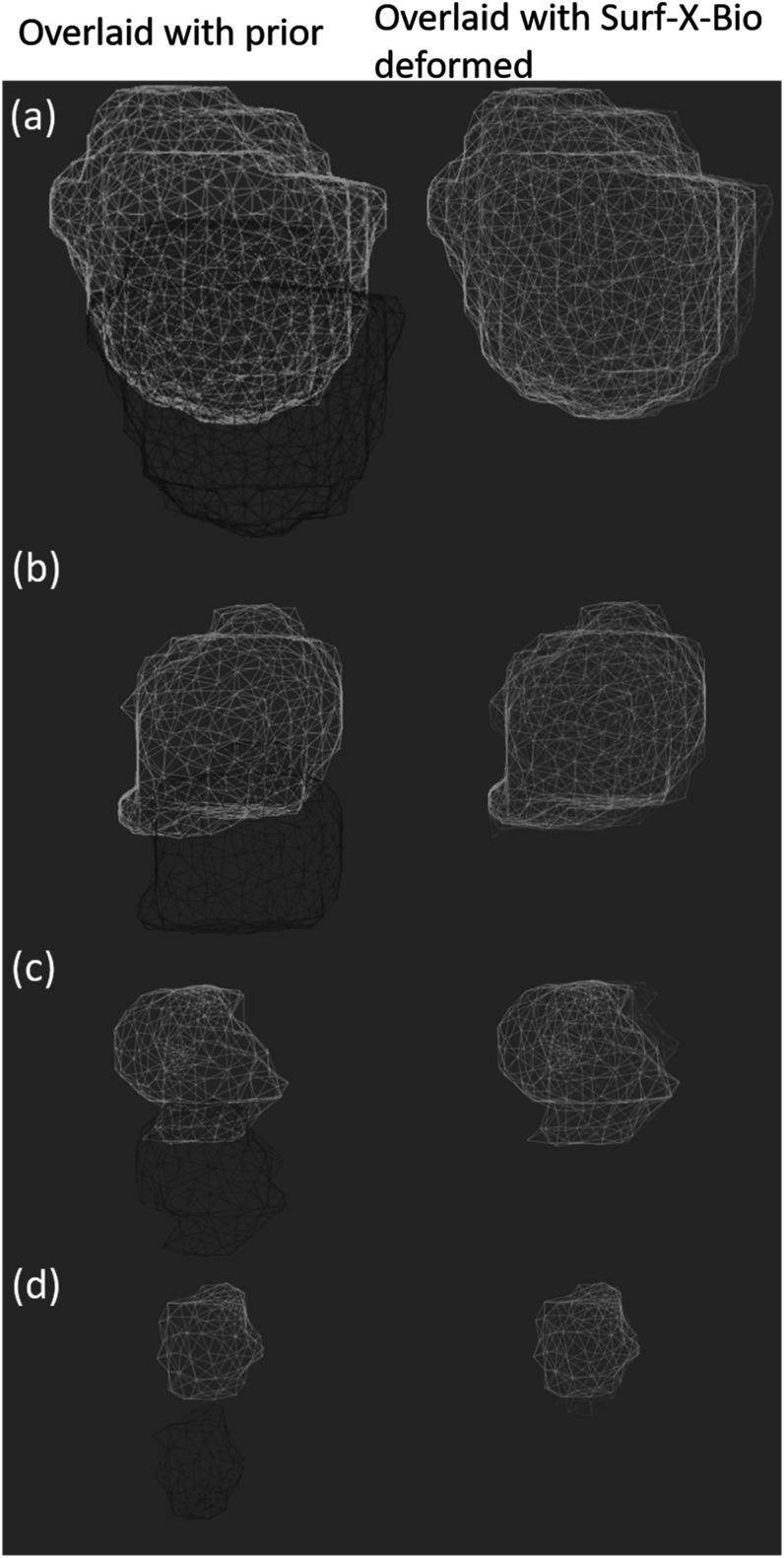
Liver tumor surface mesh overlay of Surf-X-Bio for four patient cases (a)–(d). The left and right columns show the pre- and post-registration overlays, respectively. The prior, Surf-X-Bio deformed, and ‘ground-truth’ tumor surface meshes are in red, green, and yellow colors, respectively.

Figure 6 presents liver tumor overlays for four patients in the testing set. The left column shows the overlays between the prior and ‘ground-truth’ tumor surface meshes, and the right column shows the overlays between the Surf-X-Bio deformed and ‘ground-truth’ tumor surface meshes. As shown in figure 6, good liver surface registration accuracy can translate into a good liver tumor localization accuracy after the DL-based biomechanical model for a wide range of the tumor size.

**Figure 7.**
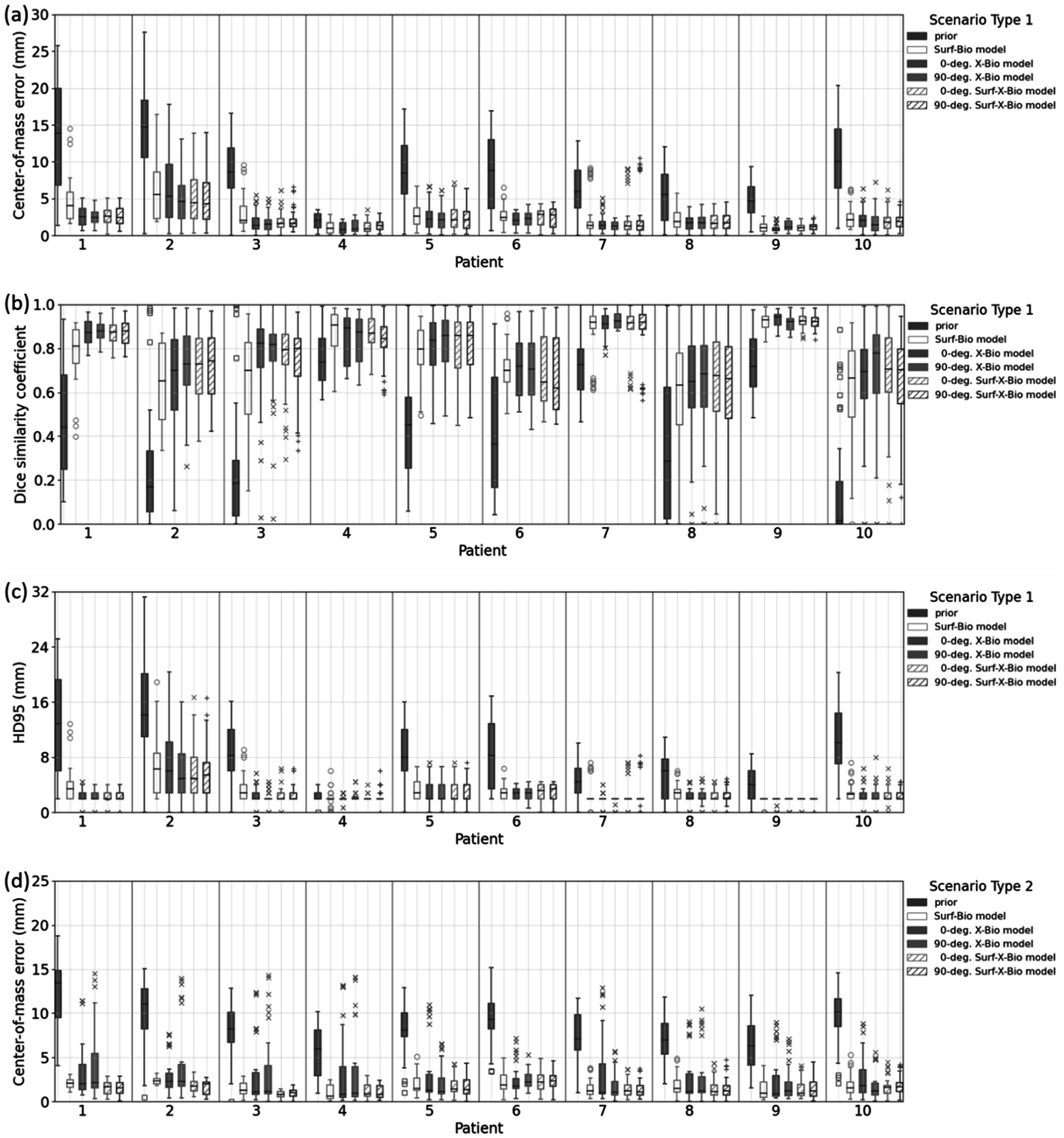
Liver tumor localization results of the four motion scenario types: (a)–(c) Type 1; (d)–(f) Type 2; (g)–(i) Type 3; and (j)–(l) Type 4. For results of each type, the upper panel shows the COME results, the middle panel shows the DSC results, and the lower panel shows the HD95 results.

Figure 7 presents the liver tumor localization results for the four types of motion scenarios. The first boxplot of each patient case shows the errors prior to the registration. The second boxplot shows the tumor localization errors of the Surf-Bio model, and the following four boxplots show the results of the X-Bio and Surf-X-Bio models at 0° and 90° projection angles, respectively. Table 3 summarizes the quantitative results of the COMEs, DSCs, and HD95s. All *p*-values of the Wilcoxon signed-rank tests between the Surf-Bio, X-Bio and Surf-X-Bio models reached the significance level (*p* < 0.05), except for the case of DSC between X-Bio and Surf-X-Bio at the 90° projection and the cases of HD95 between Surf-Bio and X-Bio at 0° and 90° projections. Overall, the Surf-X-Bio provided equal or better liver tumor localization accuracy than the others.

**Table 3.** Liver tumor center-of-mass errors (COMEs), Dice similarity coefficients (DSCs), and 95-percentile Hausdorff distances (HD95s) of the Surf-Bio, X-Bio, and Surf-X-Bio models for all tested motion scenarios. The results were presented in terms of mean and standard deviation [$\bar{X}\pm {\mathrm{SD}}$] and in terms of median and quartiles [*M* (*Q*_1_, *Q*_3_)]. The last two columns present the median differences and 95% confidence intervals between the Surf-Bio and Surf-X-bio models and between the X-Bio and Surf-X-Bio models.

			Model			Median difference	
Metric	Projection angle	Prior	Surf-Bio	X-Bio	Surf-X-Bio	Surf-Bio/Surf-X-Bio	X-Bio/Surf-X-Bio
COME (mm)	0°	8.3 ± 4.8	2.4 ± 2.1	2.5 ± 2.7	**1.9 ± 1.6**	0.4 (0.3, 0.4)	0.1 (0.1, 0.2)
				1.5 (0.8, 3.0)	**1.4 (0.8, 2.3)**		
	90°	8.0 (4.4, 11.1)	1.9 (1.1, 2.9)	2.4 ± 2.6	**1.9 ± 1.6**	0.4 (0.3, 0.4)	0.1 (0.0, 0.1)
				1.5 (0.9, 2.6)	**1.5 (0.9, 2.4)**		
DSC	0°	0.40 ± 0.29	0.78 ± 0.18	0.75 ± 0.25	**0.79 ± 0.20**	−0.04 (−0.04,−0.03)	−0.01 (−0.02, −0.01)
				0.83 (0.65, 0.93)	**0.85 (0.72, 0.92)**		
	90°	0.41 (0.12, 0.65)	0.83 (0.69, 0.91)	0.76 ± 0.24	**0.78 ± 0.20**	−0.03 (−0.03,-0.02)	0.00 (−0.01, 0.00)
				0.84 (0.68, 0.92)	**0.84 (0.70, 0.92)**		
HD95 (mm)	0°	7.9 ± 4.8	2.8 ± 1.8	2.9 ± 2.5	**2.3 ± 1.5**	1.0 (0.8, 1.1)	1.1 (0.9, 1.4)
				2.0 (2.0, 2.8)	**2.0 (2.0, 2.8)**		
	90°	7.5 (4.0, 10.2)	2.0 (2.0, 2.8)	2.8 ± 2.3	**2.4 ± 1.5**	0.9 (0.8, 1.1)	0.8 (0.6, 1.0)
				2.0 (2.0, 2.8)	**2.0 (2.0, 2.8)**		

**Figure 8.**
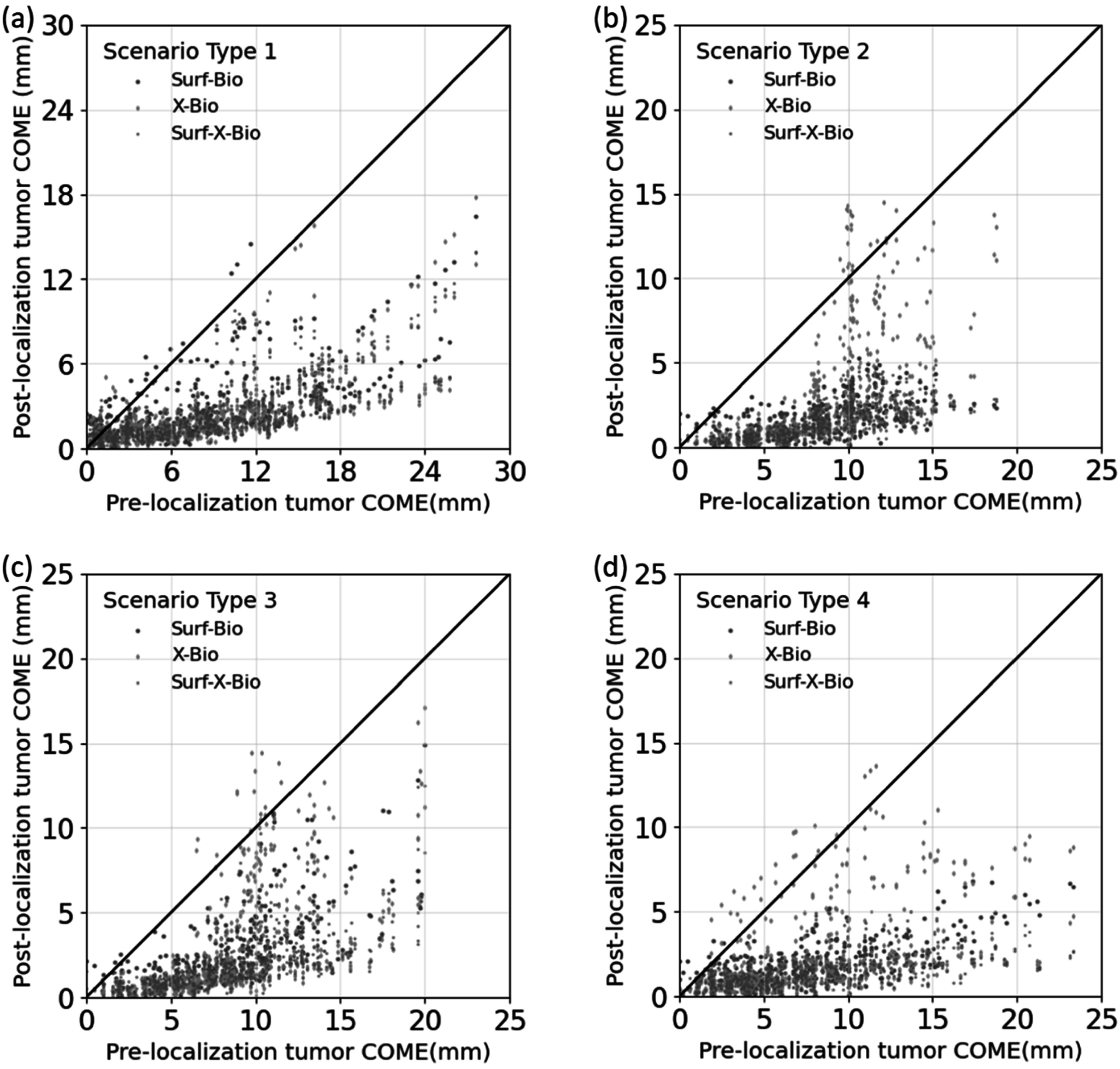
Scatter plots of liver tumor localization results. The dots are color-coded for different methods (blue: Surf-Bio; orange: X-Bio; and green: Surf-X-Bio). The *x* axis shows the liver tumor center-of-mass error (COME) prior to localization, and the *y* axis shows the residual COME after the localization. A point closer to the *x* axis indicates better localization accuracy.

To further evaluate how the three methods performed in scenarios of large motion, we further plotted figure 8 to include each localization result as a dot for all methods. For methods offering more accurate and robust results, their dots should be close to the *x* axis, particularly for ones with large pre-localization tumor COME (large motion). From figure 8, it can be observed that Surf-Bio, X-Bio and Surf-X-Bio models had more comparable tumor localization accuracy for cases with relatively small prior COMEs (≤10 mm). While for cases with large motion, Surf-X-Bio clearly outperformed the other methods. Table 4 presents the quantitative results for the normal (COMEs ≤ 10 mm) and large (COMEs > 10 mm) motion. It shows that for the larger motion group, Surf-X-Bio demonstrates a clear localization advantage.

**Table 4.** A comparison of liver tumor localization results between the Surf-Bio, X-Bio, and Surf-X-Bio models for small and large motion amplitudes. The small and large motion amplitude was stratified by the prior tumor center-of-mass error (COME). The small amplitude was defined as the prior COME ≤ 10 mm. The results were presented in terms of mean and standard deviation [$\bar{X}\pm {\mathrm{SD}}$] and in terms of median and quartiles [*M* (*Q*_1_, *Q*_3_)].

		Prior COME ≤ 10 mm			Prior COME > 10 mm		
Metric	Projection angles	Surf-Bio	X-Bio	Surf-X-Bio	Surf-Bio	X-Bio	Surf-X-Bio
COME (mm)	0°	1.7 ± 1.2	1.5 ± 1.8	1.2 ± 0.9	3.9 ± 2.6	4.4 ± 3.3	3.2 ± 2.0
		1.4 (0.8, 2.1)	1.0 (0.6, 1.7)	1.0 (0.6, 1.6)		3.0 (2.1, 5.9)	2.7 (2.0, 3.8)
	90°		1.5 ± 1.7	1.3 ± 1.0	3.0 (2.1, 4.8)	4.2 ± 3.1	3.1 ± 2.0
			1.1 (0.6, 1.7)	1.1 (0.6, 1.7)		2.9 (2.0, 5.3)	2.6 (1.9, 3.8)
DSC	0°	0.82 ± 0.14	0.85 ± 0.16	0.87 ± 0.11	0.69 ± 0.20	0.65 ± 0.25	0.73 ± 0.17
		0.87 (0.75, 0.93)	0.90 (0.79, 0.95)	0.90 (0.83, 0.95)		0.72 (0.54, 0.85)	0.78 (0.62, 0.86)
	90°		0.85 ± 0.15	0.86 ± 0.12	0.74 (0.60, 0.84)	0.66 ± 0.24	0.73 ± 0.17
			0.90 (0.80, 0.95)	0.90 (0.81, 0.95)		0.74 (0.56, 0.86)	0.78 (0.62, 0.86)
HD95 (mm)	0°	2.2 ± 1.0	2.1 ± 1.6	1.8 ± 0.9	3.9 ± 2.5	4.5 ± 3.1	3.4 ± 2.0
		2.0 (2.0, 2.0)	2.0 (2.0, 2.0)	2.0 (2.0, 2.0)		3.2 (2.0, 6.0)	2.8 (2.0, 4.0)
	90°		2.0 ± 1.6	1.9 ± 0.9	2.8 (2.0, 4.5)	4.3 ± 2.8	3.3 ± 2.0
			2.0 (2.0, 2.0)	2.0 (2.0, 2.0)		2.8 (2.0, 5.7)	2.8 (2.0, 4.0)

### 3.3. Robustness test to iso-center miscalibration between surface and x-ray imaging

The liver boundary registration and liver tumor localization results of the robustness test on the Surf-X-Bio model are summarized in tables 5 and 6, respectively. The registration errors were averaged over all motion scenarios. It can be observed that the liver surface and tumor registration errors increase as the amplitude of the iso-center offset increases.

**Table 5.** Liver boundary registration results of the robustness test. RMSE and HD95 were calculated for various magnitudes of iso-center offsets between the surface and x-ray imaging modalities. The results were presented in terms of mean and standard deviation [$\bar{X}\pm {\mathrm{SD}}$] and in terms of median and quartiles [*M* (*Q*_1_, *Q*_3_)].

		Iso-center offset (mm)		
Metric	Projection angle	0.0	1.0	2.0
RMSE (mm)	0°	1.5 ± 1.3	1.8 ± 1.3	2.4 ± 1.2
		1.0 (0.7, 1.8)	1.4 (1.1, 1.9)	2.1 (1.8, 2.6)
	90°	1.6 ± 1.3	1.8 ± 1.3	2.4 ± 1.3
		1.1 (0.8, 1.8)	1.4 (1.1, 2.0)	2.1 (1.7, 2.5)
HD95 (mm)	0°	2.3 ± 1.6	2.5 ± 1.5	3.1 ± 1.4
		1.7 (1.2, 2.8)	2.1 (1.6, 2.9)	2.7 (2.4, 3.4)
	90°	2.4 ± 1.6	2.6 ± 1.5	3.1 ± 1.4
		1.8 (1.3, 2.9)	2.1 (1.6, 3.0)	2.7 (2.3, 3.4)

**Table 6.** Liver tumor localization results of the robustness test. The results were presented in terms of mean and standard deviation [$\bar{X}\pm {\mathrm{SD}}$] and in terms of median and quartiles [*M* (*Q*_1_, *Q*_3_)].

		Iso-center offset (mm)		
Metric	Projection angle	0.0	1.0	2.0
COME (mm)	0°	1.9 ± 1.6	2.2 ± 1.6	2.9 ± 1.6
		1.4 (0.8, 2.3)	1.8 (1.2, 2.7)	2.5 (2.0, 3.3)
	90°	1.9 ± 1.6	2.2 ± 1.7	2.8 ± 1.7
		1.5 (0.9, 2.4)	1.8 (1.1, 2.6)	2.4 (1.8, 3.3)
DSC	0°	0.79 ± 0.20	0.76 ± 0.19	0.70 ± 0.19
		0.85 (0.72, 0.92)	0.82 (0.68, 0.90)	0.74 (0.60, 0.85)
	90°	0.78 ± 0.20	0.76 ± 0.19	0.71 ± 0.19
		0.84 (0.70, 0.92)	0.82 (0.67, 0.90)	0.75 (0.62, 0.86)
HD95 (mm)	0°	2.3 ± 1.5	2.6 ± 1.5	3.0 ± 1.6
		2.0 (2.0, 2.8)	2.0 (2.0, 2.8)	2.8 (2.0, 3.2)
	90°	2.4 ± 1.5	2.6 ± 1.5	2.9 ± 1.6
		2.0 (2.0, 2.8)	2.0 (2.0, 2.8)	2.8 (2.0, 3.2)

## 4. Discussion

In this study, we developed a DVF-driven framework to solve real-time liver motion and localize liver tumors. The developed Surf-X-Bio framework incorporated prior 4D-CT volumes, which provided reference liver meshes for on-board DVF-driven motion estimation and tumor localization. Meanwhile, the motion information contained within the 4D-CT set can be extracted and augmented to train deep learning neural networks to capture 3D liver boundary motion from surface imaging or x-ray imaging. The solved liver boundary motion can be propagated inside the liver via biomechanical modeling to localize the liver tumor. Surf-X-Bio cascaded three DL networks (Surf model, X model, and Bio model) to achieve accurate liver tumor localization. The Surf model was trained to learn the motion correlations between the respiratory-induced external body surface and liver boundary, and used these learnt correlations to predict liver boundary motion from on-board surface imaging. Subsequently, the X model further corrected the residual registration errors from the Surf model, using features extracted from a single on-board x-ray projection to fine-tune the liver boundary DVFs. By doing so, it was assumed that the latencies between the surface imaging and the x-ray imaging have been calibrated (Glide-Hurst *et al* 2011), such that both the surface and x-ray images correspond to the same anatomy. Finally, the population-based Bio model used the liver boundary DVFs predicted from the preceding two models as the boundary conditions to solve the intra-liver DVFs. A U-Net based model was developed to replace conventional finite-element solvers to achieve a substantial efficiency boost.

Surf-X-Bio was tested via four types of motion scenarios, which substantially deviated from the motions observed in the training stage. The mesh overlay and surface node projection show that the Surf-X-Bio model registered both the liver cranial and caudal sides accurately, and the regions close to the heart were also well registered (figure 5). The results showed that the Surf-X-Bio model was robust to the motion variations, especially for the cases with large amplitudes of deformation and translation (figure 8). It also consistently outperformed the models using surface imaging only and x-ray imaging only (figure 7, tables 3, and 4). The relative advantage and robustness of the Surf-X-Bio framework can be attributed to the introduction of the surface imaging, which provides additional information complementary to the limited FOV x-ray projection. As shown in figure 5, for a standard-sized x-ray projection (512 × 384 pixels with 0.776 × 0.776 mm^2^ per pixel), a non-negligible portion of the liver boundary nodes may not be captured by the x-ray projection and thus do not have corresponding imaging features. The loss of information is further exacerbated by the introduction of large motions in this study. Previous works showed that the single x-ray based model faced reduced accuracy in scenarios of large motion, and the solved DVFs could be trapped at local optima and failed to extract the correct motion features from the x-ray image, which were far away from the prior mesh positions due to the large motion (Nakao *et al* 2022). Using surface imaging helps to provide additional information to estimate the liver boundary motion at all liver boundary nodes through learnt internal–external correlations. The estimated liver boundary node motion by the surface model is closer to the ‘ground-truth’ and thus allows the following x-ray model to find and extract the correct motion features far away from the original mesh nodes positions. As a result, the combination of the surface imaging and the x-ray projection helps to address the ill-conditioned real-time imaging and tumor localization problem effectively. The robustness test shows that Surf-X-Bio is capable to correct the iso-center miscalibration between the surface and x-ray imaging modalities to some extent (tables 5 and 6). Since the positioning accuracies of current commercially available surface imaging systems achieve <1.0 mm (Al-Hallaq *et al* 2022), Surf-X-Bio demonstrated its capability to correct the positioning errors from the surface imaging.

Figure 7 shows a large variation over the liver tumor localization accuracy, especially for the DSC of the first type of motion scenarios. There are several potential causes for this variation: (a) the testing dataset involves cases with extensive respiratory motion and a wide range of rigid translations, and has an inherent large variability. (b) DSC is very sensitive to small-sized tumors. For example, for patients 8 and 10, the DSC of the prior tumor (without registration-driven localization) is quite extensive in range (0–1.00 and 0–0.98, respectively). The tumor diameters (approximated via a spherical geometry) of patient 8 and 10 were less than 8 mm, which was smaller than the simulated tumor motion ranges (>15 mm). The combined small tumor size and large tumor motion leads to a large variation in DSC. (c) The liver surface registration errors propagated into the Bio model and led to a wide variation of the model performance. The Bio model was a population-based model and was trained on a dataset of 24 patients. In contrast, the Surf model, the X model, and the cascaded Surf-X model were patient-specific. The Surf, X, and Surf-X models may generate patient-specific error/noise distributions in the output liver boundary DVFs that are different from those in the DVFs used to train the Bio model. Although errors/noises were added to the training liver boundary DVFs for the Bio model to enhance its robustness, it is difficult to fully cover the spectrum of noise distributions generated from the preceding models. Correspondingly, when the Bio model was cascaded with and coupled to the Surf, X, or Surf-X model, some out-of-distribution test samples will increase the variation of the liver tumor localization accuracy. Potential technique improvements like re-training and fine-tuning the Bio model based on patient-specific data and input from proceeding models remain to be investigated in future studies.

Compared with Shao *et al* (2022), in this study we reported a slightly larger mean COME of the localized liver tumor (1.9 mm versus 1.2 mm). Such a discrepancy is mostly contributed by the fact that we simulated larger deformable/rigid motion in the new study (mean pre-localization tumor COME: 8.3 mm) as compared to the pilot study (6.1 mm), with some motion scenarios deviated significantly from the training cases. Such extreme scenarios helped us to evaluate the robustness of the methods and demonstrated the advantage of combining the surface imaging with x-ray imaging. In addition, we introduced both smaller FOV and photon/electronic noises into the simulated x-ray projections, which may affect the model performance as well. In addition to noises, scatter is another major source of degradation signals for x-ray cone-beam imaging (Endo *et al* 2001, Graham *et al* 2007). We can potentially include the scatter signals into the x-ray projections as well to train a more robust model to the degradation signals. However, to incorporate scatter signals in x-ray projections for training, a Monte-Carlo (MC) based simulation algorithm is usually required (Jia *et al* 2012). It may add substantial time overhead, especially for on-the-fly motion augmentations that require the MC simulation to be performed online during training as well. Recently, there are studies using fast deep neural networks to simulate realistic cone-beam projections with degradation signals close to the MC simulation (Maier *et al* 2018, Peng *et al* 2019), which can potentially be applied to our study. Instead of directly feeding in the scatter/noise-compromised projections to train a model robust to these signals, another strategy is to pre-process the x-ray projections to remove/reduce these signals before feeding into a network trained using noise-free digitally reconstructed radiographs. Conventional hardware- or algorithm-driven techniques can be applied (Mainegra-Hing and Kawrakow 2010, Chen *et al* 2017). Deep learning-based scatter removal techniques can also be potentially applied (Lalonde *et al* 2020), which remain to be further investigated.

Liver tumor intra-fractional motion tracked via fiducial markers has been reported in literatures. Park *et al* reported ranges of liver tumor motion from treatment CBCTs of 20 patients being 2.8 ± 1.6 mm, 5.3 ± 3.1 mm, and 16.5 ± 5.7 mm for the LR, AP, and SI directions, respectively (Park *et al* 2012). Worm *et al* measured intra-fractional liver tumor motion during stereotactic body radiation therapy and found that the mean ranges of motion for 10 patients were 5.2 mm, 8.0 mm, and 16.0 mm for the LR, AP, and SI directions, respectively (Worm *et al* 2013). Xu *et al* reported mean ranges of liver tumor motion of 2.1 ± 2.3 mm, 2.9 ± 2.8 mm, and 6.4 ± 5.5 mm in the LR, AP, and SI directions, respectively (Xu *et al* 2014). Worm *et al* measured liver tumor motion during 4D-CT scans and reported mean ranges of 2.2 mm, 4.3 mm, and 11.0 mm for the LR, AP, and SI directions, respectively (Worm *et al* 2018). Excluding rigid translation, in our testing dataset the mean ranges of the liver tumor center-of-mass motion from the prior were 3.2 ± 0.9 mm, 7.0 ± 4.4 mm, and 14.1 ± 6.4 mm in the LR, AP, and SI directions, respectively. Our motion ranges were comparable to the measured ranges reported in the literatures. Moreover, in contrast to the published works which focused on the intra-fractional tumor motion only, we also included rigid motion of up to 10 mm in magnitude to simulate inter-fractional motion. Table 4 shows that for the large motion (prior COME > 10 mm), the Surf-X-Bio outperformed X-Bio and reduced the mean/median COME from 13.7/12.8 to 3.2/2.7 mm. Since large motion can sometimes occur, our results demonstrated the value of combining surface imaging with x-ray imaging for real-time liver tumor localization.

To achieve real-time motion management and adaptive radiotherapy, the overall system latency has to be kept below 500 ms such that the beam delivery system can have sufficient time to respond to real-time motion (Keall *et al* 2021). In this work, we used the deep learning-driven biomechanical model (Bio) to substantially improve the efficiency of intra-liver DVF propagation. The conventional iterative finite-element solvers took ∼30 s for each solution (Shao *et al* 2022), compared with ∼150 ms of the Bio model (which can be further accelerated through more advanced hardware). The overall latency of the proposed imaging framework was contributed by the Surf model (mean inference time: 21.7 ms), the X model (26.0 ms), and the Bio model (150.0 ms). Since the surface imaging can be captured at a high frame rate of up to 24 Hz (Al-Hallaq *et al* 2022), and the x-ray imaging can be acquired at 15 frames per second (Srinivasan, Mohammadi, and Shepherd 2014), the image acquisition latency can be achieved below 40 ms (Borman *et al* 2018). The overall imaging system latency can be controlled to ∼200 to 250 ms, fulling the temporal constraint of real-time imaging and tumor localization.

In this study, to train the patient-specific Surf and X models, all samples in the training and validation datasets were generated through the deformable and translational augmentations from the 4D-CT images. This approach yields a model tailored to each patient case to capture patient-specific motion features. It assumes that the patients’ on-board deformable motion can be well described by the motion observed from the 4D-CT. Such an assumption remains to be further validated with patient data under varies degrees of inter- and intra-fractional motions (Langen and Jones 2001, Shirato *et al* 2004, Keall, Poulsen, and Booth 2019). And if needed, inter-fractional motion models can be introduced to further augment the training/validation dataset with more deformation patterns (McClelland *et al* 2011, Park *et al* 2016). A known challenge of using real-patient data for verification, however, is the lack of ‘ground-truth’ or ‘gold-standard’ liver tumor localization data. Implanted fiducial markers can potentially be used as a reference for tumor’s rigid motion, which however cannot be used to assess tumor’s deformable motion. Physical liver phantoms that can simulate highly realistic liver deformable/rigid motion can be applied with known ‘ground-truth’ motion for reference.

## 5. Conclusion

In conclusion, we developed a deformation-driven and DL-based framework to track liver motion and localize liver tumors in 3D and real-time. Combining the complementary information from optical surface imaging and a single on-board x-ray projection, the developed Surf-X-Bio framework provides accurate liver tumor localization. It is also more robust to scenarios with large motion, as compared to models using the surface imaging or the x-ray projection alone. Boosted by a DL-based biomechanical model, Surf-X-Bio can localize liver tumors at an overall latency of less than 250 ms. It can be potentially applied towards intra-treatment tumor localization, dose calculation/accumulation, and real-time plan adaptation.

## Ethical statement

Ethical approval was obtained from the UT Southwestern Medical Center on August 31, 2013. Individual patient consent was signed for the anonymized use of the imaging and treatment planning data for retrospective analysis. The study was conducted in accordance with the principles embodied in the Declaration of Helsinki. The research was approved under an umbrella IRB protocol 082013-008 (Improving radiation treatment quality and safety by retrospective data analysis). This is a retrospective analysis study and not a clinical trial. No clinical trial ID number is available.
